# “Babies born early?” - silences about prematurity and their consequences

**DOI:** 10.1186/s12978-018-0594-4

**Published:** 2018-09-12

**Authors:** Maria J. O. Miele, Rodolfo C. Pacagnella, Maria J. D. Osis, Carina R. Angelini, Jussara L. Souza, José G. Cecatti

**Affiliations:** 1 Department of Obstetrics and Gynecology, School of Medical Science, University of Campinas, Campinas, São Paulo Brazil; 2 Department of Pediatrics, School of Medical Science, University of Campinas, Campinas, São Paulo Brazil; 3 Jundiai Medical School, São Paulo, Brazil; 4 São Leopoldo Mandic Medical School, São Paulo, Brazil

**Keywords:** Preterm birth, Qualitative research, Health literacy, Parto prematuro, Investigación cualitativa, Literatura saludable, Nascimento prematuro, Pesquisa qualitativa, Alfabetização em Saúde

## Abstract

**Background:**

The principal aim of this study was to understand how communication between parents and health professionals concerning prematurity occurs, from delivery to admission to the neonatal Intensive Care Unit.

**Methods:**

This is an exploratory, descriptive study with a qualitative methodology. Data were collected using tape-recorded and Focal Groups technique interview with mothers of premature newborns and health professionals involved in caring for preterm infants, at southeast Brazil.

**Results:**

The word “premature” was not said or heard during prenatal care. From the narratives, it was observed that there was a lack of information available to pregnant women about preterm birth, failure in medical care regarding signs and symptoms reported by pregnant women, and lack of communication between the medical teams, mothers and family during delivery and Neonatal Intensive Care Unit (NICU) admission.

**Conclusion:**

There is a fine line between born too soon and die too soon, that increases stress, fear and distance impacting negatively over communication between mothers and health professionals during antenatal care, childbirth and NICU admission.

## Resumen

**Introducción:**

El objetivo principal de este estudio fue comprender cómo se produce la comunicación entre los padres y los profesionales de la salud con respecto a la prematuridad, desde el nacimiento hasta la admisión en la Unidad de Cuidado Intensivo neonatal.

**Métodos:**

Este es un estudio exploratorio, descriptivo-cualitativo. Los datos fueron recolectados mediante la grabación de entrevistas y el uso de la técnica de Grupos Focales con madres de recién nacidos prematuros y profesionales de la salud involucrados en el cuidado de bebés prematuros, en el sudeste de Brasil.

**Resultados:**

La palabra “prematuro” no se dijo ni escuchó durante la atención prenatal. A partir de las narrativas, se observó falta de disponibilidad de información sobre el nacimiento prematuro, la ausencia de atención médica en relación con los signos y síntomas notificados por las mujeres embarazadas y la falta de comunicación entre los equipos médicos, las madres y la familia durante el parto y la admisión a la Unidad de Cuidado Intensivo Neonatal (UCIN).

**Conclusión:**

Hay una línea muy fina entre nacer demasiado pronto y morir demasiado pronto, lo que aumenta el estrés, el miedo y la distancia impactando negativamente sobre la comunicación entre las madres y los profesionales de la salud durante la atención prenatal, el parto y la admisión en la UCIN.

## Resumo

**Introdução:**

O objetivo principal deste estudo foi compreender como ocorre a comunicação entre pais e profissionais de saúde sobre a prematuridade, desde o parto até a internação na Unidade de Terapia Intensiva Neonatal.

**Método:**

Trata-se de um estudo exploratório, descritivo e com metodologia qualitativa. Os dados foram coletados por meio de entrevista gravada, utilizando a técnica de Grupos Focais com mães de recém-nascidos prematuros e profissionais de saúde envolvidos no atendimento de prematuros no sudeste do Brasil.

**Resultados:**

A palavra “prematuridade” não foi dita ou ouvida durante o pré-natal. Das narrativas, observou-se a falta de informações disponível às gestantes sobre parto prematuro, falhas na assistência médica quanto aos sinais e sintomas relatados pelas gestantes e falhas na comunicação entre as equipes médicas e as mães e familiares durante o parto e Admissão em Unidade de Terapia Intensiva Neonatal (UTIN).

**Conclusão:**

Há uma linha tênue entre nascer ou morrer antes da hora, o que aumenta o estresse, o medo e a distância, afetando negativamente a comunicação entre mães e profissionais de saúde durante o pré-natal, o parto e a internação na UTIN.

## Plain English summary

Prematurity kills one child every 30 s and affects around 10% of pregnancies worldwide. This causes an enormous impact over families and health professionals. Dialogue with the patient represents a window of opportunity to listen (to) and understand information. Hence, any difficulty of dialogue between health professionals and mothers need to be explored.

In order to explore how the dialogue between families and health professionals occurs concerning preterm birth, we invited mothers of premature newborns and health professionals involved in the care for preterm infants for a focus group.

The question we had in mind was: “How do they talk about signs and symptoms of risk for preterm born, the childbirth and prematurity? What is it important to say?” Furthermore, whose responsibility is to give that information to the parents, how they do that and “Have the parents understood all information?”

In conclusion, the current study gave us the opportunity to understand the relationships between those involved in the care of preterm babies. Families need information, and this must be provided by health professionals who have responsibility for identification of risk in antenatal care. How well the information is transmitted information depends on the between parents and health professionals.

## Background

The preterm birth rate is estimated at around 10% worldwide. In Brazil, the percentage of preterm births is slightly above 12% of all births. Complications resulting from preterm birth account for 35% of neonatal deaths and is the second most common direct cause of death in children aged 5 years or less [1–3]. Sequelae due to preterm birth (PTB) may influence both the child and family members throughout life [4, 5].

Brazil offers a mix of health facilities providing levels of specialized care thru public health system called Unified National Health System. These Health Care System recommendations in the Humanization Program in Prenatal Care and Birth which proposes a welcoming atmosphere for accompanying and educative activities, to help women solve their doubts during prenatal care until delivery [6]. According to the Prenatal Health Care Manual, the prenatal period is a time when any abnormality in pregnancy may be perceived and damage minimized, moreover, make a link with the maternity hospital [7]. In the national survey “Birth in Brazil”, was described the prenatal support at prenatal care. The survey showed that most women (74,6%) had prenatal care at health public of which 73,1% had 6 or more appointments. Although 88,4% was attended by the same health professional, only 58.7% has advised about the maternity hospital there should search for at the delivery, and 16.2% had difficult to find a health service in the birthing time [8].

The low quality of prenatal care, however, is strongly associated with (PTB). An empathetic attitude of the staff can strength the dialogue between doctor and patient may positively influence patient adherence to treatment [9]. Likewise, when the health team feels compassion for the mother’s situation, it becomes easy to feel their vulnerability. Compassion is essential to develop trust and attentive communication between the health team and parents [10]). Communication is the essential element during the clinical consultation. When listening and information are lacking during prenatal care, the possibilities of appropriate treatment may also fail [11].

Thus yet, communication is only complete when it is included in the process of health literacy. Health literacy is the capacity to understand given information, make correct use of guidance and be able to make decisions about body or suggested management [12]. When the dialogue is adequately associated with mother’s needs, rises chances of success during prenatal care to prevent preterm birth and its consequences.

This study aimed to explore the communication between health staff and mothers when a baby is born prematurely. Our mean idea was listening to those women and with the result of this listening will give an opportunity to understand where the gaps are. We assumed that pregnant women lacked knowledge about the physiological and metabolic risks of preterm birth. The comprehension may indicate a pathway for better communication in the health service, creates a tool for future actions for prevention and minimizes the damages of PTB.

## Methods

An exploratory qualitative design was chosen to address relevant questions about little known or explored contents and clarify gaps during this trajectory, indicating new paths and action strategies for health communication [13].

### Data collection

This study was carried out in a maternity hospital with more than 30 years’ experience in women’s and children’s health. It is located in Campinas (state of São Paulo), one of the largest metropolitan regions of Brazil. The hospital is a high-risk obstetrics and neonatology referral center, with over 3000 deliveries per year As a complex of reference in maternal and neonatal care this maternity school, covers a metropolitan region and 41 neighboring municipalities, besides other cities in the State of São Paulo and other states. This condition attracts patients from many regions searching for specialized care.

Owing to its interactive characteristic, the Focus Group technique was chosen. Group dynamics may bring out the opportunity to encourage all participants to express their opinions [14, 15]. To delimit the number of participants, the Focus Group criterion according to Krueger was used [16], with the ideal number between 5 and 8 participants.

Initially, our proposal was to listen to the narratives of two distinct groups. One group was composed of mothers of preterm newborn infants and the other consisted in NICU professionals involved in prenatal care, delivery and preterm infant care from the institution who were working during the study period.

Given the aims and design of the study, an intentional sample with a focus group was used, with participants in each group. Only one focus group was chosen due to information obtained during preliminary analysis, which was sufficient for study aims.

The main researcher initiated the process of acculturation and familiarization in the Neonatal Intensive Care Unit (NICU) previously to know the people, groups and individuals relevant to the study. During the entire period, a “Field Diary” (FD) was also constructed as a data collection instrument. The FD contained a description of the trajectory and observations of the researcher [17].

Data collection took place from April to June 2015 with the participation of the main researcher and a psychologist with experience in FG. To carry out the FG, a Debate Syllabus was used, based on assumptions that guided the study.

The aim of the questions was to know the steps of caring for preterm infants. We used a focus group guide that included the following questions: “What do parents know and what do they need to know about preterm birth?,” , “What do women know about the risk of having preterm birth in antenatal care?”, “What is important for parents to know or be instructed at admission in the NICU?”, “What do parents know about pre-mature children at Hospital Discharge?”, “In your opinion, how could the information be transmitted in a useful and clear way?”. Following the central theme, the same trigger questions were framed to the focus group of professionals and parents. FG discussions were held in a dedicated room to ensure the privacy of the participants. FG were audio-recorded after participants presented and signed the consent term.

### Data analysis

It was not the intention of this article to make an exhaustive analysis of the theme. However, focus was on the discussion of specific categories, and each subtitle represented a distinct axis of data analysis.

For data analysis, the approach used was a content thematic analysis of narratives found in FG discussions [18], with the first stage was the transcription, speed reading and material organization. The researcher listened to recordings from each group separately, respecting the chronology of the event. After identifying voices and silences expressed by informants, recordings were transcribed. In the second phase, themes that emerged from the set of conversations in the FG were identified. From these themes, the core meaning was sought, which then established categories of analysis. Parts of the text pertaining to each category were decoded and classified. To define categories, in addition to the relevance given by informants or repetition of content in conversations, there were meetings between researchers to form a consensus. Then, internal validation was made by peers, psychologists, sociologists, doctors and nurses, participants of an academic study group in qualitative research. The COREQ checklist was used to assure the adequate report of this study [19].

## Results

An invitation was made and repeated to the professional group, to turn it technically possible on the day of group formation. Two FGs were formed (Table 1), one with seven mothers of preterm infants in the NICU (MFG), all arrived from external prenatal care and their infants were born between 24 and 33 gestational weeks, all searched for the Maternity from small cities. Although it was technically possible on the day of group formation, none of the nursing staff members participated. The conversations and perceptions of this team could improve our understanding of some barriers of communication between parents and professionals that were emphasized in the FG. Nevertheless, the current study was an exploratory analysis to the topic.

**Table 1:** Characterization of the sample according to the Focus Groups age/time works, occupation/profession and deliveries

Maternal Focus Group			
Name	Occupation	Deliveries	Age (years)
Angélica	Artisan	Multiparous	39
Hortencia	Shop worker	Primiparous	34
Jasmim	Administrative assistant	Primiparous	29
Melissa	Manicure	Multiparous	24
Yasmin	Production leader	Primiparous	27
Rosa	Salesperson	Primiparous	23
Violeta	Receptionist	Primiparous	26
Professional Focus Group			
Name	Works at NICU	Profession	
Atena	1 year	Psychologist	
Ártemis	6 years	Neonatologist	
Hera	11 years	Neonatologist	
Iris	3 years	Resident	
Persephone	3 years	Resident	
Selene	3 years	Resident	

In our study was wide feminine hegemony in the composition of both groups, turning into a silence any masculine opinion about the theme. Regarding the PFG, the health teams of the NICU are mostly composed of female members making these sample a clear reproduction.

The choice of only one focus group from each sample was derived from the information obtained, which during the preliminary analysis was sufficient for the study objectives.

Content analysis has indicated four crucial moments that chronologically had been reflected the meaning that guided the need to give and seek information on preterm birth. The first period refers to prenatal care, when there was no risk of preterm birth. The second period refers to delivery and birth. The third refers to NICU admission and lastly the hospital discharge. Based on these specific periods, we initiated the process of categorization, listening to contents that emerged in each group. The criterion for definition of categories was the relevance emphasized by informants, or the repetition of content in conversations. Whereas those moments, we present the categories and the contents side by side, to demonstrate how each group experienced the process of communication and their meaningful about preterm birth.

**Fig. 1:**
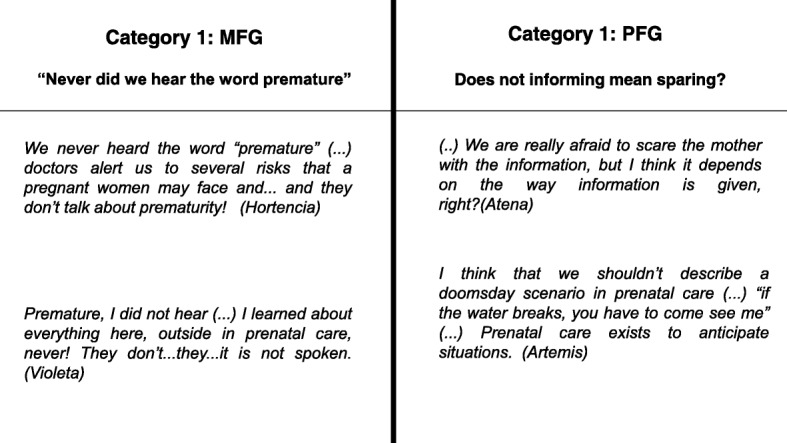
The two sets of categories identified in the FGs and their contents regarding information concern premature birth during the prenatal care

The first question posed was: “what do women know about preterm birth at the beginning of pregnancy?” The MFG reported that the possibility of having a preterm infant was not considered, neither by the pregnant women nor prenatal care doctors. In the other hand, the perception of the PFG was from NICU viewpoint and, seeing from that perspective, they understood the physicians attend to warn the mother of the possibility of preterm birth without frightening the pregnant woman (Fig. 1).

**Fig. 2:**
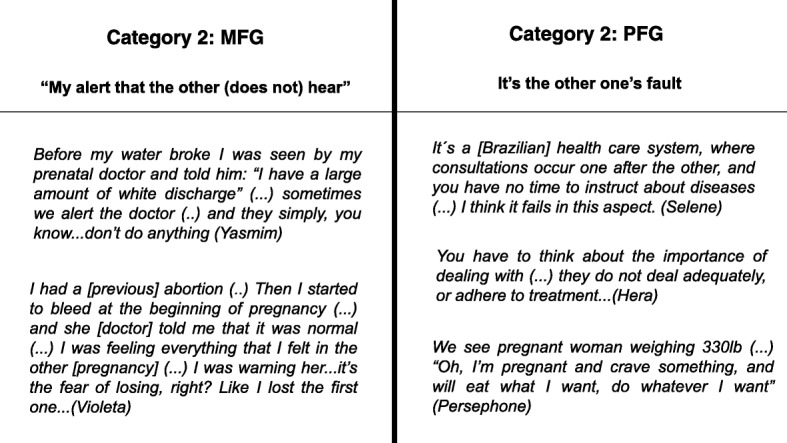
The two sets at categories identified in the FGs and their report about the prevention during the prenatal care

Groups were also asked about what pregnant women knew or should be informed about the prevention preterm birth. However, after premature birth, both groups seem to seek one another for late answers to difficulties encountered in communication and information (Fig. 2).

### Delivery

**Fig. 3:**
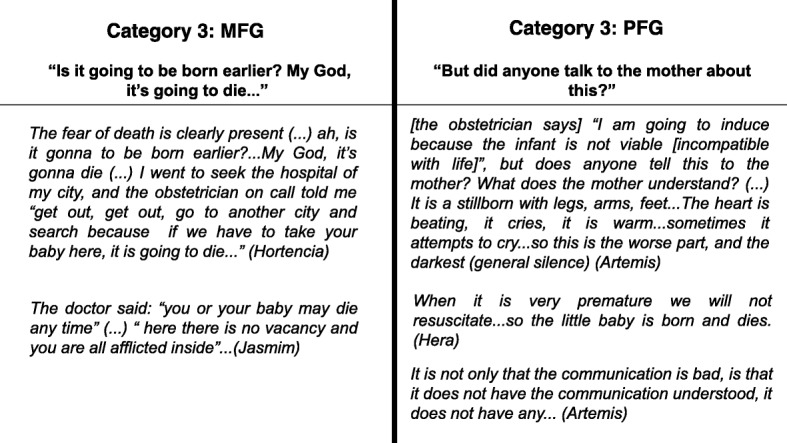
The two sets of categories identified in the FGs and their contents about support and understand regarding the childbirth

Both groups were asked about what mothers knew about the delivery labor preterm. To the MFG, information came accompanied by the possibility of losing their babies or their own life. Therefore, the first contact of those women with preterm birth was the risk of death. For women in the PFG, the major difficulty was communication between teams that work in the same location, despite different foci and aims. The loss of a baby impacts the entire team. Giving the news in a clear and adequate way to a woman is a great challenge to all. The perceptions expressed in conversations and categories of both groups were similar (Fig. 3).

### NICU

**Fig. 4:**
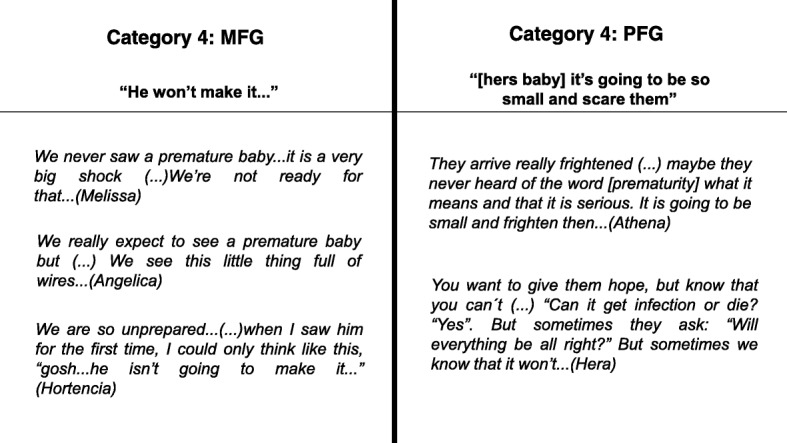
The sets of categories and contents related to the first contact between parents and premature newborn at the NICU admission

On admission, the trigger question was “what is important for parents to know or be instructed during hospitalization of the infant?” It motivated the debates in each group. Diverse contents emerged from the groups, giving sequence to categories in a chronological order. The issue raised by the MFG reflected the first visit of the mother to the infant in the NICU, the shock of seeing her baby surrounded by tubes and devices, and the fear of losing the newborn child. The perception of shock and maternal lack of preparation was also clear among those in the PFG. Both groups shared the same perception and feelings regarding this matter (Fig. 4).

**Fig. 5:**
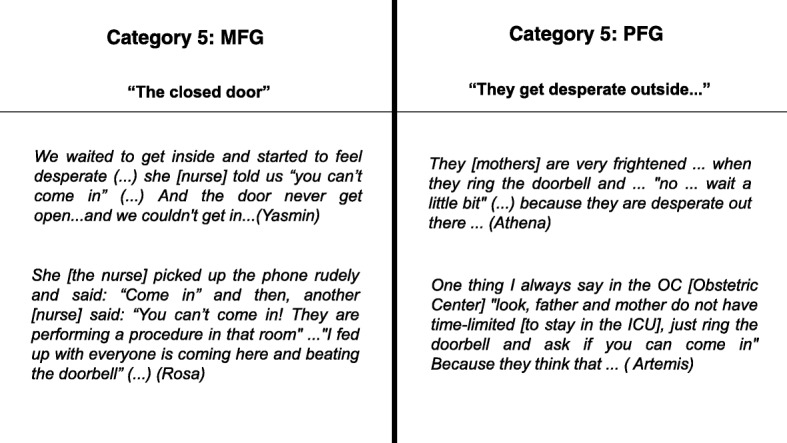
The sets of categories and contents reported by the groups regarding their perception about the mother’s access to their child

The PFG revealed the perception of “despair” of mothers and participants and mentioned that they encouraged them to exert their role in the NICU. Nevertheless, they recognized that the pressures imposed by the NICU dynamic contributed to the creation of barriers between professionals and parents (Fig. 5).

Mothers are forced to block their most basic desire to protect their babies. The MFG believe that the power in the NICU lies with the nursing team. Access to the infant and doctors must pass through these nurses. This feeling is so strong that FD records indicated that these mothers did not talk about any impediment created by the nursing team, not even in the group offered by the NICU team to resolve doubts, where these women participated. However, in this same group, women called themselves “the prisoners.” When asked about the reason for such a comparison, women reported that “prison guards were the health professionals, mothers felt as though they were prisoners of their infants, which were prisoners of the NICU.”

**Fig. 6:**
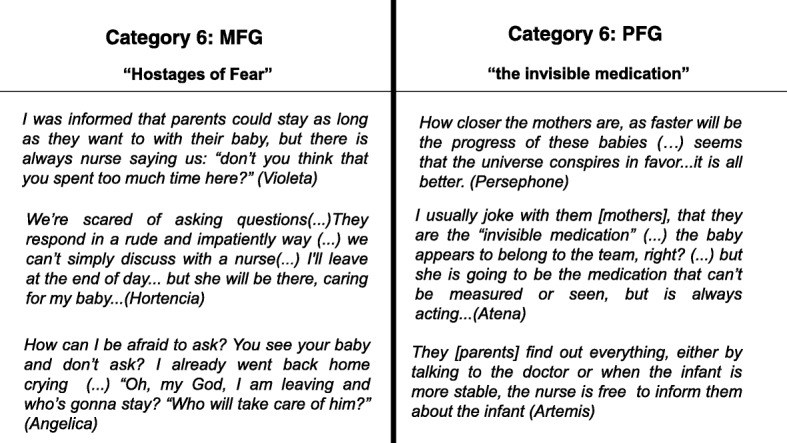
The sets of categories and the description of emergent contents related to NICU and the distance of opinions and perceptions

In the other hand, the PFG thinks all the information is made available to the mothers and they have free access to prescription. These professionals saw no reasons for mothers to avoid asking any questions to the nursing team or medical team, when required (Fig. 6).

### Hospital discharge

**Fig. 7:**
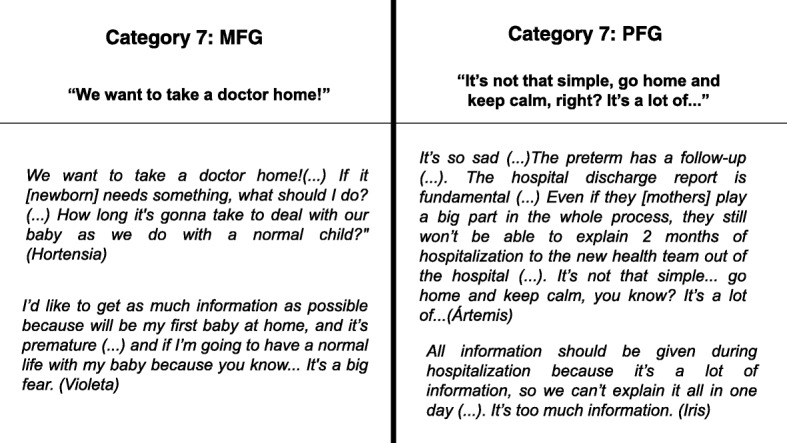
The sets of categories and contents referred by the groups demonstrating the distance of communication about parent understanding

For the MFG, news about hospital discharge of the infant transformed a deeply desired moment into a frightening event. The reason was because they felt unprepared to be mothers of preterm infants, without medical help. The PFG reported difficulty in explaining so much information. Due to the complexity, they were concerned about the continuity of treatment and need for communication with the new team that will provide follow-up care to the infant after hospital discharge (Fig. 7).

## Discussion

Although prematurity represents 15 million births worldwide, the word “premature” in this study was not said or heard during prenatal care. Through the women voices, were expounded the difficulty to approach the preterm theme. During antenatal care, maternal signs and symptoms should alert obstetricians to potential maternal clinical conditions which may lead to preterm birth [20, 21]. However, without the adequate time to listen to complaints of the patient and provide proper health care according to the level of risk, prevention is likely to fail. A study of 457 pregnant women investigated the type information on health and educative actions that was acquired during prenatal care. These women declared that they did not receive necessary information during prenatal visits that lasted three or less minutes. This result opposes the primary proposal of prenatal care, which is to obtain information and education as tools to make the woman feel empowered, thus contributing to a safer pregnancy [11].

The MFG reported their perception and concern with diverse unknown signs and symptoms, and when women sought more information, they were not heard. On the perspective of PFG, failures and misunderstandings were attributed to an overburdened health system with a high patient demand. Kangasniemi et al. [22], evaluated maternal perceptions over their health during pregnancy. Despite reporting a greater responsibility for their pregnancy and baby’s health, those women also reported they did not receive the necessary information and instructions about needs of changes in their health during prenatal care [22].

The understanding should come from both sides. Health staff comprehending the difficulty of each patient and adapting the dialogue. Patients must be more active and take initiative to ask for more explanation when they do not understand the information. In common, a recent study investigated the influence of health assistance at prenatal care related with parity, socioeconomic level, age and maternal schooling. Which concluded that only the maternal schooling was positively associated with the quality of prenatal care. The authors indicated that interventions with trained health teams can enhance communication and improve their understanding to help women [23].

The capacity to give and understand information is linked to education. Low health literacy has created a dual communication barrier between doctors and patients. In addition to a lack of comprehension of the conversations, the patient is placed in a passive condition inhibiting further search for information [24]. In Brazil, a higher level of school education is associated with the capacity to prevent and treat diseases, lower morbidity/mortality rates and increased number of prenatal visits and is an important factor for health protection.

Women from the MFG received two bad news at the same time. One was that their lives and that of their babies were at risk. The second was to obtain urgent access to a maternity hospital with a structure to give the necessary support for both. The threat of death during childbirth turned the most important moment in the woman’s life reduced into fear and desperation.

Delay in obtaining assistance was attributed to the organization of Brazilian Health System, which categorizes the pregnant woman to different health services, according to the level of complexity. This implies in transporting the pregnant woman great distances, when she lives in small municipalities and requires specialized care [25] .

The PFG reported the absence of communication between obstetricians and neonatologists. It is two teams acting in the same space with the same patient, but they do not exchange any information or share in decision-making. That scenario seems to reflect the importance of building a bridge to dialogue. In agreement Iams et al. [26], indicated that the lack of communication between teams hinders optimal care. To solve the problem, the authors suggest solutions involving communication skills [26]. Studies indicate that communication between obstetricians and pediatricians is a valuable tool in caring for the newborn infant and the mother. Therefore, it is important to understand that childbirth is a multidisciplinary event. Training the team should involve communication and mutual support [27, 28].

After th impact of the first maternal visit to the newborn infants, the groups observed the barriers faced by mothers when accompanying their infants in the NICU. Furthermore, there was the physical distance from their homes to the hospital. The hospitalized infant seems to belong to the NICU team more than to the mother. Finding the door to the NICU closed, illustrates only part of this scenario which reproduces the feeling of incapacity to protect the baby and exert mothering.

The meaning of pregnancy for a woman begins many years before when she still a child playing with a doll in her arms. However, when something interrupts the desired course of pregnancy, the responsibility for this “failure” lies on female shoulders, generating feelings of guilt [25]. Premature birth is like a book with pages missing, represents the rupture of a situation in which the woman is no longer pregnant, but has still not become a mother, since the child is under medical care and not her own. Rosa [29] wrote about the feeling of not belonging to a known place. From those mothers perspective, only an invisible place is reserved, between a moment before and the promise of future, dwelling in a point unknown between familiar sides [29].

This symbolic image conveys the inadequacy of the MFG at a time when they are not patients nor the medical team, neither can be the mothers that they have been dreaming to be. Because, the child was born but they did not become mothers. The first moment between mother and child become inside the NICU, it is a special moment and should be facilitated by the health care team, so the mothers can exert mothering. Even though this child is hospitalized, excessive interference should be avoided when mother and child are together [30]. That condition turns a woman in a “ICU Mother,” feeling apart of the child and the information about their health condition.

Franck and Axelin [31] compared the perception of parents, nursing team and doctors regarding the care and information that family members receive in the NICU They found a disconnection of perceptions with physicians and nurses believing in an adequate support to the parents at most of the time. In contrast, parents felt excluded from caring for the infant and in decision-making. Apart from that, they felt distant from their child and without emotional support [31]. The same difficulty of access and lack of participation in decision-making related to the infant was observed in another Brazilian study [30]. This type of support has been increasingly attempted by neonatology teams. Various initiatives are designed to reduce distances and place parents as main characters of the process. The “kangaroo-mother” method was an initiative also implemented in the maternity where the current study was conducted [32].

Although hospital discharge was deeply desired, taking care of a preterm infant generated insecurity and concern for the MFG. Loneliness and helplessness emerged, feelings that they had often dreaded. Furthermore, their reports echoed the desire to maintain the same bonds that had previously maintained them prisoners of devices and constant medical presence. Similar results were obtained in a study evaluating what care meant to the mother after preterm infant discharge. Controversial feelings such as fear and anxiety were experienced, in addition to love and affection at being able to take care of their infants [33]. Mothers also reported the need to receive training to be competent during NICU stay. This would reduce the insecurity at the time of infant discharge. The PFG reported greater concern with the volume of information necessary both at discharge and post-discharge care. These contents reinforce the need to provide information in a progressive and continuous manner during hospital stay. The mothers’ suggested pamphlets or booklets about signals and symptoms at prenatal care, and lecture at the waiting room about difficulties in self-care. For the PFG, reported that the first step should be a better communication between health team through periodical meetings and more contact with mothers at prenatal care. They also proposed more mother’s autonomy during some babies’ care procedures, with less intervention by the nursing team. Those suggestions are in agreement with Pedrini et al. [34], who recommends that mothers have printed information to support conversations during prenatal care, for better counseling. Moreover, neonatologists an obstetricians conducted counselling separated and was not found any information about if they received training in communication skills [34].

An important limitation of this study was that we were unable to hear all the professionals involved in care. The conversations and perceptions of this team could improve our understanding of some barriers of communication between parents and professionals that were emphasized in the FG. Nevertheless, the current study offers an exploratory analysis to approach the topic.

The viewpoint of nurse technicians may be further investigated in future studies and was not essential to the aim of the study which was to explore the communication between professionals and family members about preterm birth.

The current study taught us the opportunity to understand the distance between actors involved from the time of conception to the arrival of the baby is proportional to the capacity to give and receive information. Comprising that the effort should always be sought for patients and their newborn well-being, we propose a diagram of horizontal communication between health team and family for motherly empowerment as the choice for flow of information approaching during specific moments.

## Conclusions

There is a thin line between life and death on prematurity that block the communication during antenatal care, increases stress around childbirth and brings fear during NICU hospitalization. All these things negatively impact mothers and health professionals.

Understanding the meaning of prematurity can open a door for dialogue, with listening beyond what is said but the comprehension by the patient and doctor. However, to support parent’s vulnerability health staff must work together in a unison speech. In order to promote that change, we propose a strategic equidistant approach for each specific time. During delivery, the mother-child contact should be preserved, and horizontality chosen for flow of information.

## Abbreviations

FD: Field Diary
FG: Focus Group
ICU: Intensive Care Unit
MFG: Mothers Focus Group
NICU: Neonatal Intensive Care Unit
PFG: Professional Focus Group
PTB: Preterm Birth
